# Reciprocal regulation of TLR2-mediated IFN-β production by SHP2 and Gsk3β

**DOI:** 10.1038/s41598-017-07316-3

**Published:** 2017-07-28

**Authors:** Jin Hee Park, Ryeojin Ko, Soo Young Lee

**Affiliations:** 1 Department of Life Science, Ewha Womans University, Seoul, 120-750 Korea; 2 The Research Center for Cellular Homeostasis, Ewha Womans University, Seoul, 120-750 Korea

## Abstract

Toll-like receptor 2 (TLR2) mediates the innate immune response to bacterial lipopeptides and peptidoglycans by stimulating the production of inflammatory cytokines. However, the mechanisms by which TLR2 signaling regulates type I interferon (IFN)-β production are poorly understood. Here, we identified Src homology 2-containing protein tyrosine phosphatase 2 (SHP2) as a negative regulator of TLR2-induced IFN-β production. Pharmacological inhibition or reduced expression of SHP2 potentiated TLR2 agonist-mediated IFN-β transcription and STAT1 activation, whereas overexpression of SHP2 impaired IFN-β transcription and STAT1 activation. SHP2 physically associated with the glycogen synthase kinase 3β (Gsk3β) in an agonist-dependent manner. Gsk3β positively regulates transcription of IFN-β following TLR2 stimulation by inhibiting the phosphorylation of SHP2. SHP2 inhibited the transcriptional activity of IRF-1 and IRF-8 at the IFN-β promoter. Remarkably, IRF-1 and IRF-8 are recruited to the IFN-β promoter in a SHP2 phosphatase activity-dependent manner. These findings provide insight into the mechanisms by which SHP2 and Gsk3β work together to modulate TLR2-mediated IFN-β production in macrophages.

## Introduction

Toll-like receptors (TLR) play a critical role in the early innate immune response to pathogens by recognizing pathogen-associated molecular patterns (PAMPs) and are involved in sensing endogenous danger signals^1, 2^. TLR2, which is expressed on monocytes, mature macrophages and dendritic cells, and mast cells, specifically recognizes components of Gram-positive bacteria, including bacterial lipoproteins, lipomannans and lipoteichoic acids^3^. TLR2 can form a heterodimer with either TLR1 to recognize triacylated lipopeptides, such as the synthetic ligand Pam_3_CSK_4_, or TLR6 to recognize diacylated lipopeptides like MALP-2^1, 3–5^. The dimerization of these TLRs allows for recognition of a more specific and wider array of microbial components^6^.

Upon stimulation with TLR2 ligands, MyD88 recruits IL-1 receptor-associated kinase-4 (IRAK-4) to TLR2 through interaction of the death domains of both molecules. IRAK-1 is activated by phosphorylation and associates with TRAF6, thereby activating the IKK complex and leading to activation of MAP kinases and NF-κB^3, 7–9^. These signaling pathways are critical for TLR2-mediated pro- and/or anti-inflammatory cytokine production. Tollip and IRAK-M interact with IRAK-1 and negatively regulate TLR-mediated signaling pathways^10, 11^. Recent data indicate that localization of TLR2 ligands within endosomal compartments regulates TLR2-mediated induction of type I interferons (IFNs)^12–15^, suggesting a possible role of endocytic pathways in TLR2 signaling. However, the regulatory factor and/or signaling pathways that lead to TLR2-mediated type I IFN induction are unclear.

Src homology 2 domain-containing protein tyrosine phosphatase 2 (SHP2) is an evolutionarily conserved protein tyrosine phosphatase with a widespread expression pattern^16^. It contains two N-terminal SH domains, a classic protein-tyrosine phosphatase domain at the C-terminal end and two important tyrosine resides (Tyr542 and Tyr580) in the C-terminal tail^16, 17^. SHP2 positively regulates cytokine and growth factor signaling, but negatively regulates the activation of T and B lymphocytes and IFN-γ signaling^18–21^. These regulatory roles of SHP2 have been proposed to occur in either a phosphatase activity-dependent or -independent manner; e.g., SHP2 inhibits TLR3- and TLR4-activated IFN-β production in a phosphatase activity-independent manner by binding to TANK-binding kinase 1 (TBK1)^21^. SHP2 activity is regulated by several molecules, ZAP70 and PKA, in multiple signaling pathways^22–25^.

In the present study, we demonstrate that SHP2 and Gsk3β reciprocally regulate TLR2-mediated IFN-β production, thereby identifying SHP2 and Gsk3β as a negative and a positive regulator, respectively, of the TLR2 signaling pathway.

## Results

### SHP2 is a negative regulator of IFN-β induction by TLR2 ligand

**Figure 1:**
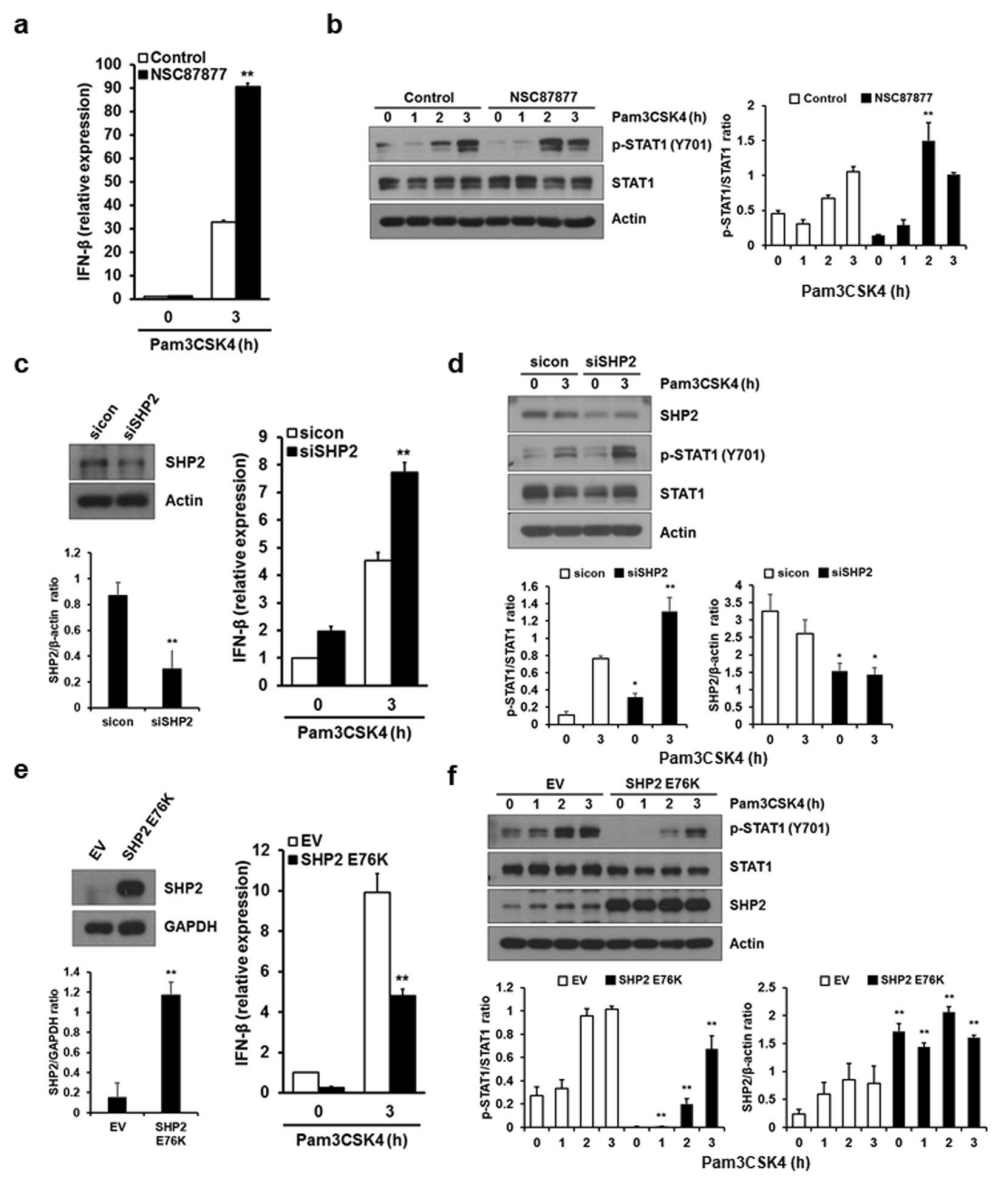
SHP2 negatively regulates IFN-β induction by TLR2 ligands. (**a**,**b**) BMDMs were pretreated with 50 μM SHP2 inhibitor, NSC87877, for 1 h and stimulated with 1 μg/ml Pam3CSK4 for the indicated times. IFN-β mRNA expression was determined by real-time PCR and the values were normalized to β-actin mRNA expression (**a**). STAT1 phosphorylation and expression levels were determined by Western blotting (**b**). (**c**,**d**) BMDMs were transfected with 50 nM control siRNAs (sicon) or SHP2-specific siRNAs (siSHP2) and stimulated with 1 μg/ml Pam3CSK4 for the indicated times. IFN-β mRNA levels were measured as demonstrated in (**c**). Western blotting was performed to determine the phosphorylation and expression levels of STAT1 and SHP2 protein levels (**d**). (**e**,**f**) BMDMs were infected with a retrovirus expressing pMX-Puro (EV) or constitutively active SHP2 (SHP2 E76K). After puromycin selection, 1 μg/ml Pam3CSK4 was applied for the indicated times. Experiments were performed as described in (**c**,**d**). Representative Western blots and quantification (shown in the bar graph) of indicated proteins/control ratio in the lysates of cells are shown in (**b**,**c**,**d**,**e**,**f**). Data represent the average of triplicate samples ± SD and are representative of at least three experiments. Statistical analyses were done using Student’s *t*-test (*P < 0.05, **P < 0.01). The uncropped images are in Supplementary Fig. S9.

SHP2 negatively regulates TRIF-dependent pro-inflammatory cytokines and type I IFN induction in TLR3 and TLR4 signaling^21^. Although SHP2 did not affect TLR2-mediated pro-inflammatory cytokine production, no previous report investigated whether SHP2 regulates TLR2-mediated IFN-β induction. TLR2 agonist Pam3CSK4 could trigger IFN-β expression at both mRNA and protein levels in macrophages (Supplementary Fig. S1). We next examined the effects of the SHP2 inhibitor NSC87877 on IFN-β expression induced by the Pam3CSK4 in macrophages. Pharmacological inhibition of SHP2 potentiated Pam3CSK4-induced IFN-β mRNA expression (Fig. 1a). SHP2 undergoes rapid phosphorylation of the tyrosine residue Y542 upon Pam3CSK4 stimulation (Supplementary Fig. S2), suggesting that a TLR2 signaling pathway involving SHP2 regulates IFN-β expression. In the canonical type I IFN-induced signaling pathway, IFN-β activates Janus kinase 1 and tyrosine kinase 2, which phosphorylate the cytoplasmic transcription factors signal transducer and activator of transcription 1 (STAT1) and STAT2^26, 27^. Phosphorylation of STAT1 upon Pam3CSK4 treatment reached a maximum at 3 h and declined thereafter (Supplementary Fig. S2). Thus, we examined the effect of SHP2 inhibition on the phosphorylation of STAT1 and observed that NSC87877 augmented Pam3CSK4-induced STAT1 phosphorylation (Fig. 1b). Pretreatment with a protein transport inhibitor, Brefeldin A, completely abolished STAT1 phosphorylation (Supplementary Fig. S3). Moreover, macrophages from mice deficient in the IFN α/β receptor (*IFNAR* ^−/−^) failed to phosphorylate STAT1 (Supplementary Fig. S3). These data suggest that IFN-β signaling induced by Pam3CSK4 directly mediates STAT1 phosphorylation.

Similar results were obtained when we used small interfering RNA (siRNA) to suppress endogenous SHP2 expression in macrophages. Silencing of SHP2 markedly increased Pam3CSK4-induced IFN-β mRNA expression and STAT1 phosphorylation (Fig. 1c,d). In contrast, IFN-β induction as well as STAT1 phosphorylation upon Pam3CSK4 stimulation was significantly decreased when a constitutively active mutant of SHP2 (SHP2 E76K) was overexpressed in macrophages (Fig. 1e,f). These results indicate that SHP2 negatively regulates TLR2-mediated IFN-β induction and the secondary response to IFN-β, i.e. STAT1 activation, in a phosphatase activity-dependent manner.

### Gsk3β positively regulates TLR2-mediated IFN-β induction

**Figure 2:**
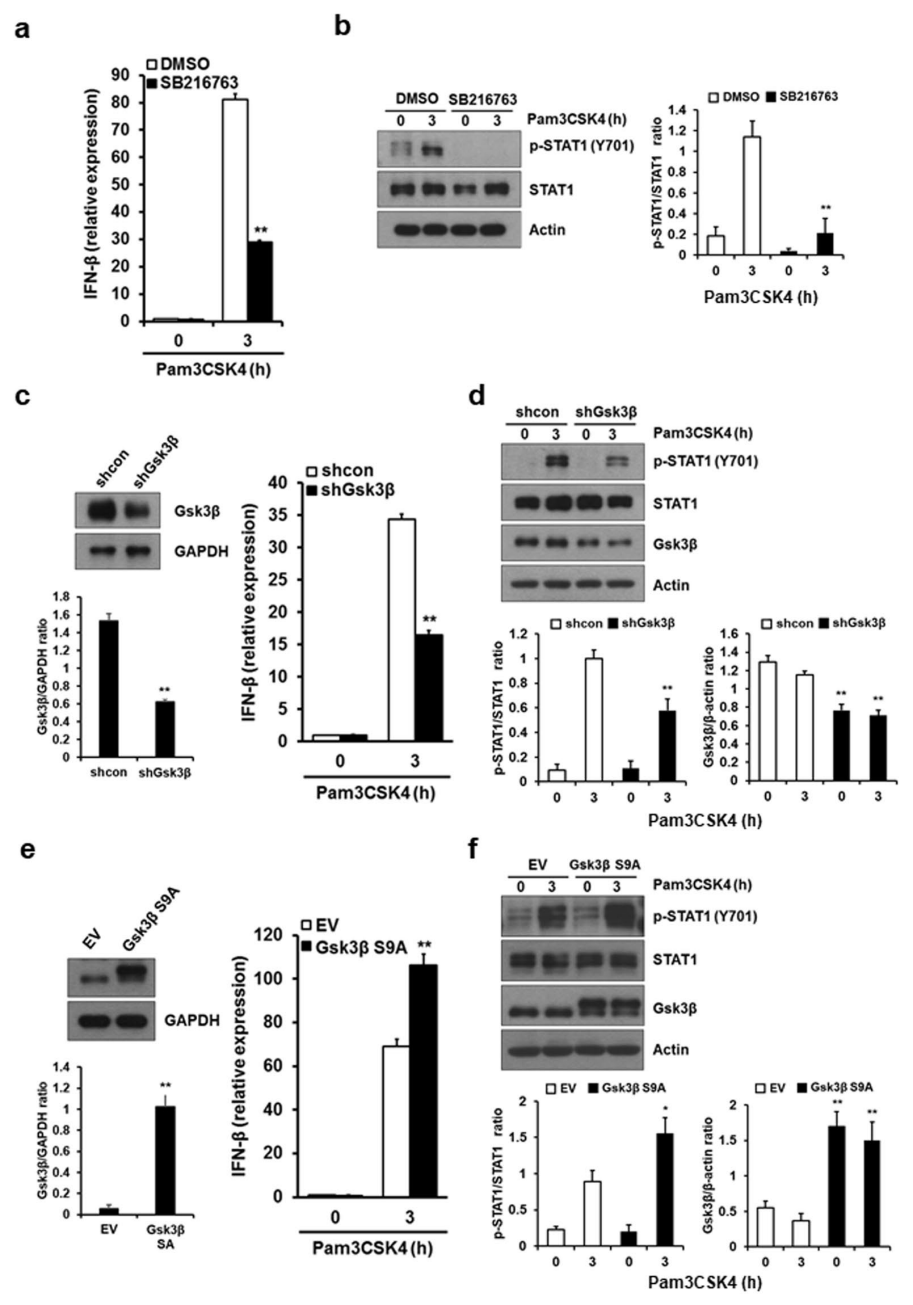
Gsk3β is involved in IFN-β induction upon TLR2 stimulation. (**a**,**b**) BMDMs were pretreated with 5 μM Gsk3β inhibitor, SB216763, for 30 min and stimulated with 1 μg/ml Pam3CSK4 for the indicated times. IFN-β mRNA levels were measured by real-time PCR (**a**). STAT1 phosphorylation and expression levels of STAT1 were determined by Western blotting (**b**). (**c**,**d**) BMDMS were infected with a retrovirus expressing control shRNA (shcon) or Gsk3β-specific shRNA (shGsk3β) as in Fig. 1c. Expression of IFN-β mRNA was measured as in (**c**). STAT1 phosphorylation and expression levels and Gsk3β protein levels were determined as in (**d**). (**e**,**f**) BMDMs were infected with a retrovirus expressing pMX-Puro (EV) or constitutively active Gsk3β (Gsk3β S9A). The experiment was performed as described in (**c**,**d**). Representative Western blots and quantification (shown in the bar graph) of indicated proteins/control ratio in the lysates of cells are shown in (**b**,**c**,**d**,**e**,**f**). Data represent the means of triplicate samples ± SD and are representative of at least three experiments. Statistical analyses were carried out using Student’s *t*-test (*P < 0.05, **P < 0.01). The uncropped images are in Supplementary Fig. S10.

IFN-β production by TLR4-stimulated macrophages is negatively regulated by a serine/threonine kinase, Gsk3β^28^. Thus, it would be interesting to investigate whether the Gsk3β signaling axis is critically involved in TLR2-mediated IFN-β induction. Unlike TLR4 signaling where IFN-β production is negatively regulated by Gsk3β, Gsk3-inactivated macrophages treated with the Gsk3 inhibitor SB216763 showed a marked decrease in IFN-β mRNA levels as compared with cells stimulated with Pam3CSK4 alone (Fig. 2a). To confirm the regulatory role of Gsk3β in TLR2-mediated IFN-β expression, the effect of Gsk3 inhibition on Pam3CSK4-induced STAT1 phosphorylation was observed. As shown in Fig. 2b, Gsk3 inhibition resulted in a significant decrease in STAT1 phosphorylation in Pam3CSK4-stimulated macrophages (Fig. 2b). Further, similar results were observed in macrophages silenced by a short hairpin RNA (shRNA) specific for Gsk3β, shGsk3β (Fig. 2c,d). Conversely, overexpression of the constitutively active Gsk3β mutant (Gsk3β S9A) in macrophages markedly elevated IFN-β mRNA expression and STAT1 phosphorylation after Pam3CSK4 treatment (Fig. 2e,f). Together, these data indicate that Gsk3β positively regulates TLR2-induced IFN-β induction.

### Gsk3β interacts with SHP2 and counteracts the inhibitory effect of SHP2

**Figure 3:**
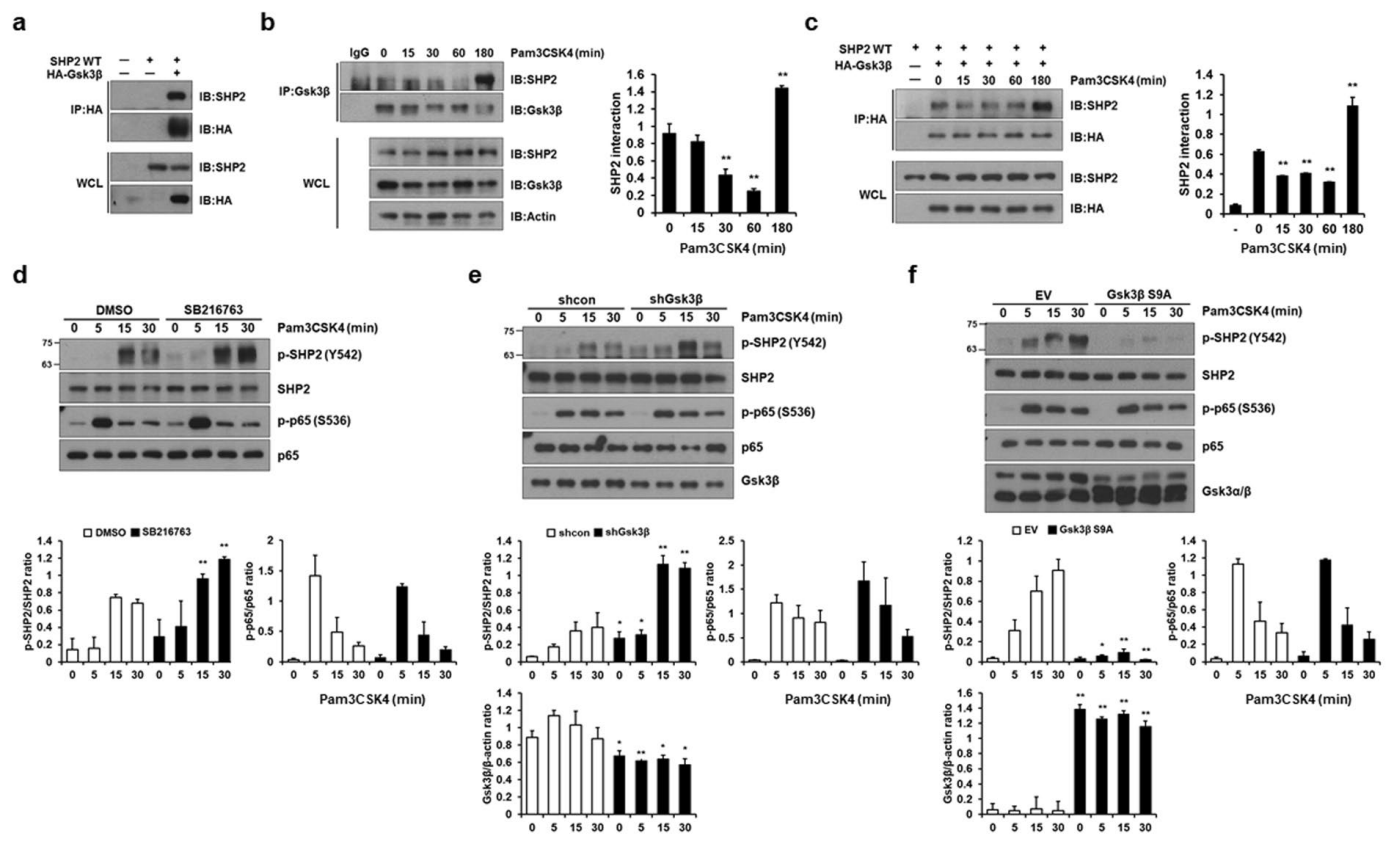
Gsk3β binds to SHP2 and prevents SHP2 phosphorylation upon TLR2 stimulation. (**a**) HEK293T cells were transfected with SHP2 WT or HA-Gsk3β expression vectors in the indicated combinations. Co-immunoprecipitation was performed with anti-HA antibody followed by Western blotting with the indicated antibodies. (**b**) BMDMs were stimulated with 1 μg/ml Pam3CSK4 for the indicated times and immunoprecipitated with IgG or an anti-Gsk3β antibody. Western blotting was performed using the indicated antibodies. (**c**) HEK293-TLR2 cells were co-transfected with the HA-Gsk3β and SHP2 WT expression vectors as indicated. After 48 h, the cells were stimulated with 1 μg/ml Pam3CSK4 for the indicated times and immunoprecipitated using an anti-HA antibody. Western blotting was performed with the indicated antibodies. (**d**) BMDMs were pre-incubated with 5 μM Gsk3β inhibitor, SB216763, for 30 min and treated with 1 μg/ml Pam3CSK4 for the indicated times. Phosphorylation and expression levels of SHP2 and p65 were detected by Western blotting. (**e**) BMDMS were infected with a retrovirus expressing control shRNA (shcon) or Gsk3β-specific shRNA (shGsk3β). After puromycin selection, cells were stimulated with 1 μg/ml Pam3CSK4 for the indicated times. Phosphorylation and expression levels of SHP2, p65, and Gsk3α/β were detected as described in (**d**). (**f**) BMDMs were infected with a retrovirus expressing pMX-Puro (EV) or constitutively active Gsk3β (Gsk3β S9A). After puromycin selection, cells were stimulated with 1 μg/ml Pam3CSK4 for the indicated times. Representative Western blots and quantification (shown in the bar graph) of indicated proteins/control ratio in the lysates of cells are shown in (**b**,**c**,**d**,**e**,**f**). Data represent the means of triplicate samples ± SD and are representative of at least three experiments). The uncropped images are in Supplementary Fig. S11.

To understand the differential regulatory mechanisms controlled by SHP2 and Gsk3β in Pam3CSK4-mediated IFN-β induction, we initially investigated the interaction between SHP2 and Gsk3β. When HEK293T cells were co-transfected with SHP2 and HA-Gsk3β constructs, SHP2 co-immunoprecipitated with HA-Gsk3β, indicating that SHP2 interacted with Gsk3β in mammalian cells (Fig. 3a). We next investigated whether SHP2 endogenously interacts with Gsk3β. We observed that SHP2 endogenously interacted with Gsk3β in macrophages in response to Pam3CSK4 at the 3 h time point (Fig. 3b). The same results were obtained in HEK293-TLR2 cells when cells overexpressing SHP2 wild-type and HA-Gsk3β were immunoprecipitated with HA-Gsk3β after TLR2 stimulation (Fig. 3c).

Considering what we knew of the interaction between SHP2 and Gsk3β, we next questioned whether SHP2 and Gsk3β reciprocally regulate each other’s activity during TLR2 signaling. Gsk3 inhibition with SB216763 resulted in significantly increased phosphorylation of SHP2 after Pam3CSK4 stimulation, although p65 phosphorylation was not altered (Fig. 3d). Similarly, Gsk3β knockdown with shRNA in macrophages significantly increased Pam3CSK4-induced SHP2 phosphorylation (Fig. 3e). In contrast, Gsk3β S9A-overexpressing macrophages showed a marked inhibition of SHP2 phosphorylation (Fig. 3f). It is interesting to note that neither overexpression of SHP2 E76K, a constitutively active mutant of SHP2, nor SHP2 inhibition with SHP2-specific siRNA altered Pam3CSK4-mediated Gsk3β phosphorylation (Supplementary Fig. S4). These results suggest that Gsk3β may act upstream of SHP2 to prevent the inhibitory function of SHP2 in TLR2-mediated IFN-β induction.

### IRF1 and IRF8 are involved in TLR2-mediated IFN-β induction

**Figure 4:**
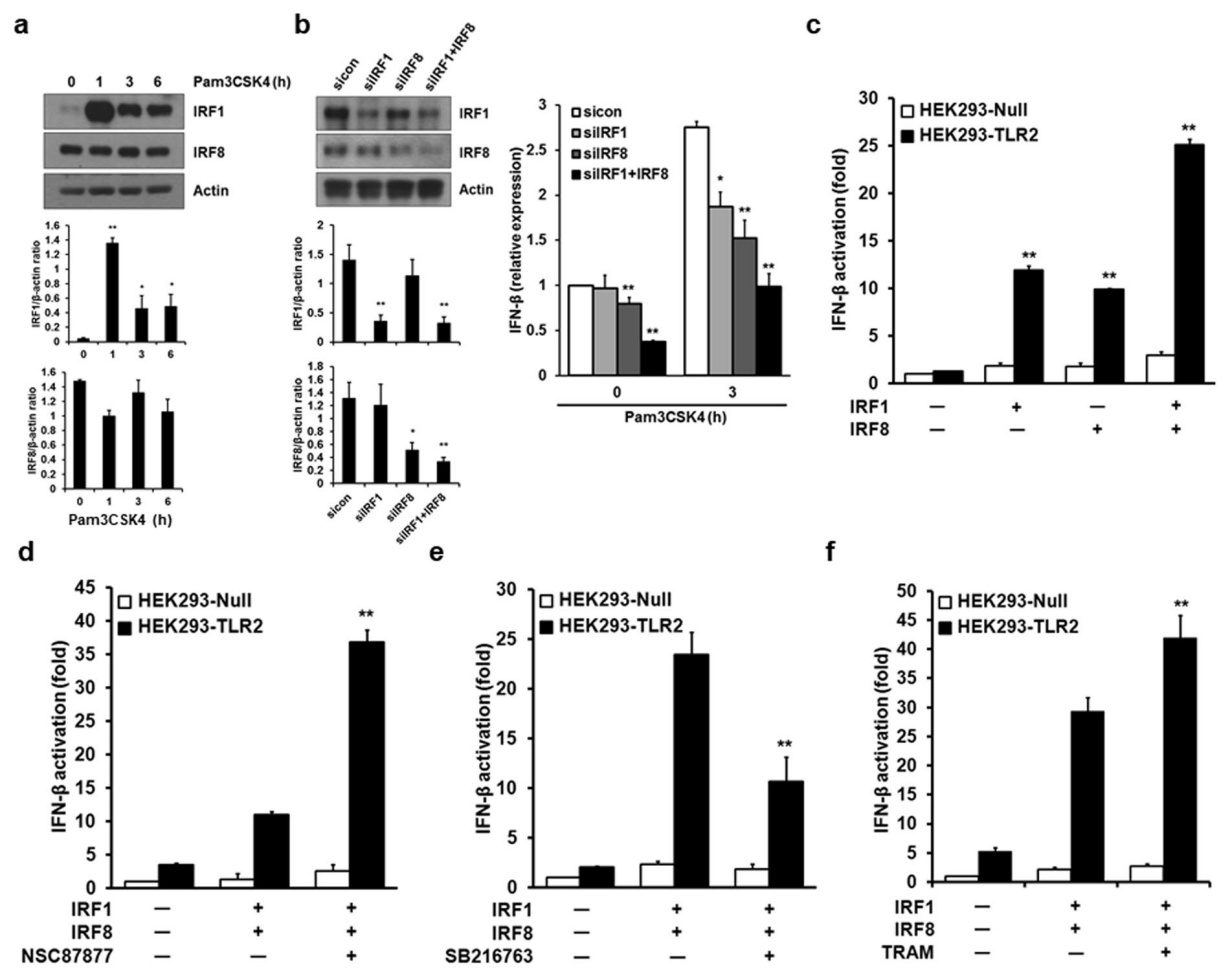
IRF1 and IRF8 are involved in IFN-β induction by TLR2 ligands. (**a**) BMDMs were stimulated by 1 μg/ml Pam3CSK4 for the indicated time. IRF1 and IRF8 levels were detected by Western blotting. (**b**) 50 nM control siRNAs (sicon) or IRF1- and IRF8-specific siRNAs (siIRF1 and siIRF8) were transfected into BMDMs and cells were stimulated with 1 μg/ml Pam3CSK4 for 3 h. IRF1 and IRF8 protein levels were determined by Western blotting, and IFN-β mRNA expression was measured by real-time-PCR. (**c**) HEK293-Null or -TLR2 cells were co-transfected with an IFN-β luc promoter and an IRF1 or IRF8 expression vector alone or in the indicated combinations. After 48 h, cells were stimulated with 1 μg/ml Pam3CSK4 for 1 h. Firefly and Renilla luciferase activities were determined, and data were normalized to the activity of Renilla luciferase. (**d**) HEK293-Null or -TLR2 cells were co-transfected with the IFN-β luc promoter and IRF1 and IRF8 expression vectors in the indicated combinations. After 48 h, cells were pretreated with 50 μM NSC87877 for 1 h and stimulated with 1 μg/ml Pam3CSK4 for 1 h. The experiment was performed as described in (**c**). (**e**) As in (**d**), except that the cells were pretreated with 5 μM SB216763 for 30 min. (**f**) HEK293-Null or -TLR2 cells were co-transfected with the indicated combinations as in (**d**). Representative Western blots and quantification (shown in the bar graph) of indicated proteins/control ratio in the lysates of cells are shown in (**a**,**b**). Data represent the means of triplicate samples ± SD and are representative of at least three experiments. Statistical analyses were carried out using Student’s *t*-test (*P < 0.05, **P < 0.01). The uncropped images are in Supplementary Fig. S12.

The transcription factors responsible for type I IFN induction are members of the interferon regulatory factor (IRF) family^29^. IRF1 is known as a transcription factor involved in TLR2-mediated IFN-β induction^13^. Because it has been reported that IRF8 is a substrate of SHP2 and SHP2 negatively regulates TLR3 signaling^30–32^, we questioned whether IRF8 together with IRF1 is involved in TLR2-mediated IFN-β induction. We first examined the expression patterns of IRF1 and IRF8 following treatment of macrophages with Pam3CSK4. As shown in Fig. 4a, IRF1 was rapidly induced by Pam3CSK4 stimulation up to the 1 h time point and declined thereafter, but IRF8 expression remained constant over time. We next determined whether IRF1 and IRF8 are required for TLR2-induced IFN-β expression using IRF1- and IRF8-specific siRNAs to silence those genes. Silencing of IRF1 or IRF8 markedly inhibited endogenous expression of the *IFNB1* gene in macrophages (Fig. 4b). *IFNB1* gene expression was significantly reduced when both IRF1 and IRF8 were simultaneously silenced by siRNAs. Moreover, co-expression of IRF1 and IRF8 in HEK293-TLR2 cells significantly increased IFN-β promoter activity compared to IRF1 or IRF8 alone (Fig. 4c).

Because a signaling pathway involving SHP2 and Gsk3β was important for TLR2-mediated type I IFN induction, we next examined the effect of SHP2 and Gsk3β on IRF1- and IRF8-induced IFN-β promoter activity. Pharmacological inhibition of SHP2 significantly enhanced activation of the IFN-β promoter induced by IRF1 and IRF8 (Fig. 4d), whereas treatment with a Gsk3 inhibitor markedly inhibited IRF1/IRF8-mediated activation of the IFN-β promoter (Fig. 4e).

It has shown previously that TRIF-related adaptor molecule (TRAM)^14, 33, 34^, a sorting adaptor not only for TLR2, but also for TLR4, is required for the production of TLR2-dependent type I IFN response at the endosome. Our results agree with those findings in that TRAM adaptor but not TRIF^35^ is required for Pam3CSK4-mediated IFN-β induction via an endosome-dependent pathway (Figs 4f and S5).

### SHP2 inhibits the recruitment of IRF1 and IRF8 to the *IFNB1* promoter

**Figure 5:**
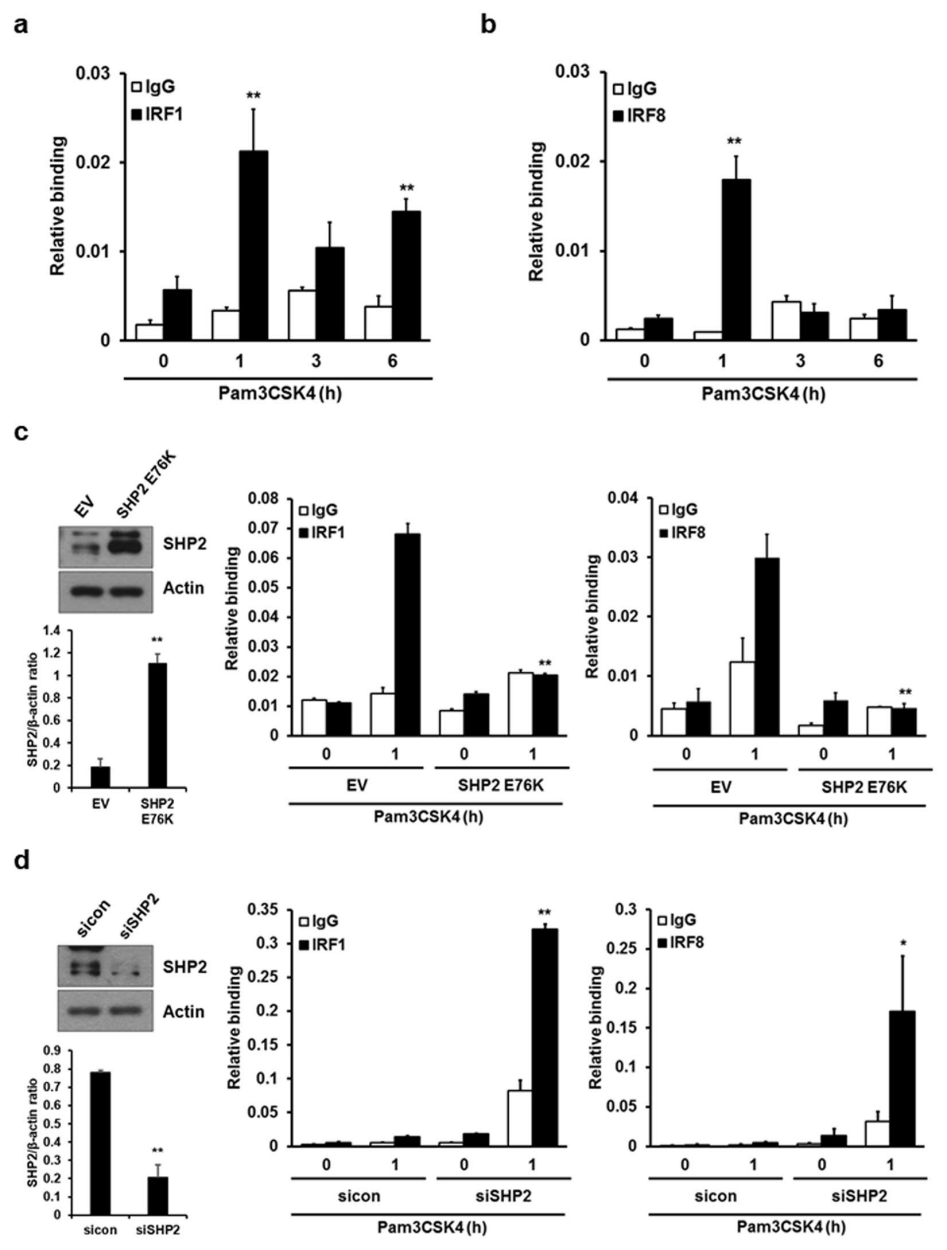
SHP2 affects the DNA binding activity of IRF1 and IRF8. (**a**,**b**) BMDMs were stimulated with 1 μg/ml Pam3CSK4 for the indicated times. ChIP analysis was performed for the IFN-β promoter using an anti-IRF1 or -IRF8 antibody or control IgG. IRF1 and IRF8 binding activity was determined by real-time PCR. (**c**) BMDMs were infected with a retrovirus expressing pMX-Puro (EV) or constitutively active SHP2 (SHP2 E76K). After puromycine selection, cells were treated with 1 μg/ml Pam3CSK4 for 1 h and ChIP analysis was performed as described in (**a**). The SHP2 expression level was analyzed by Western blotting. (**d**) 50 nM control siRNAs (sicon) or SHP2-specific siRNAs (siSHP2) were transfected into BMDMs and cells were stimulated for 1 h with 1 μg/ml Pam3CSK4. ChIP analysis was performed as described in (**a**). Expression levels of SHP2 were analyzed as described in (**c**). Representative Western blots and quantification (shown in the bar graph) of indicated proteins/control ratio in the lysates of cells are shown in (**c**,**d**). Data represent the means of triplicate samples ± SD and are representative of at least three experiments. Statistical analyses were carried out using Student’s *t*-test (*P < 0.05, **P < 0.01). The uncropped images are in Supplementary Fig. S13.

Recently, Tailor *et al*. reported that IRF8 binds directly to the *IFNB1* locus^35^; our data also showed that IRF1 and IRF8 regulate TLR2-mediated IFN-β induction at the transcriptional level. Therefore, we examined recruitment of IRF1 and IRF8 to the *IFNB1* promoter using chromatin immunoprecipitation (ChIP) assays after stimulation of TLR2 in macrophages. Chromatin from macrophages treated with Pam3CSK4 for various time intervals or from untreated controls was used for immunoprecipitation with an anti-IRF1 or anti-IRF8 antibody, and precipitated DNA encompassing the *IFNB1* gene promoter was then assayed by real-time PCR (Fig. 5a,b). The results showed that recruitment of IRF1 and IRF8 increased within 1 h of Pam3CSK4 stimulation. Binding of IRF8 drastically decreased with somewhat faster kinetics than that of IRF1 but was sustained thereafter.

Next, to determine whether binding of IRF1 and IRF8 is regulated by SHP2, the SHP2 E76K mutant was overexpressed in macrophages. The results showed that SHP2 E76K inhibited recruitment of IRF1 and IRF8 to the *IFNB1* promoter (Fig. 5c). In contrast, knockdown of SHP2 with siRNA remarkably enhanced the binding of IRF1 and IRF8 to the *IFNB1* promoter (Fig. 5d). These data demonstrated that SHP2 negatively regulates TLR2-dependent induction of IFN-β by affecting the binding of IRF1 and IRF8 to the *IFNB1* promoter.

## Discussion

Type I IFNs are pleiotropic cytokines well recognized for their role in the induction of a potent antiviral response that is an essential part of the host defense system^36–38^. Although early investigations focused on the antiviral properties of type I IFNs, recent findings indicated that type I IFN signaling also plays a key role in the defense against bacterial infection^39–41^. In this regard, we found that SHP2 negatively regulates bacterial lipoprotein mimic Pam3CSK4-mediated IFN-β induction in macrophages. We used SHP2 siRNA to suppress endogenous SHP2 expression in primary mouse bone marrow-derived macrophages. Pam3CSK4-induced IFN-β expression was increased, and this effect correlated with the suppression of endogenous SHP2 expression. The negative role of SHP2 in Pam3CSK4-induced type I IFN signaling was confirmed by both pharmacological inhibition and overexpression of SHP2.

Previous studies revealed that Gsk3β differentially regulates TLR-mediated induction of pro- and anti-inflammatory cytokines, including IL-1β, IL-12p40 and IL-10, by affecting the nuclear amount of p65 and CREB interacting with CBP^42, 43^. In addition, Gsk3β is critical for virus-triggered IRF3 activation and IFN-β production, and its functions in those regards are independent of its kinase activity^44, 45^. Instead, Gsk3β mediates oligomerization and auto-phosphorylation of TBK1, a kinase responsible for IRF3 phosphorylation after viral infection. In this study, we found that Gsk3β, unlike SHP2, promoted Pam3CSK4-induced type I IFN expression by binding to and controlling SHP2 in the TRAM-dependent endocytic pathway involving TLR2 signaling.

Overexpression of Gsk3β markedly inhibited Pam3CSK4-mediated SHP2 phosphorylation. Conversely, shRNA-mediated knockdown of Gsk3β increased SHP2 phosphorylation. Consistently, pharmacological inhibition of Gsk3 produced impaired SHP2 phosphorylation after Pam3CSK4 stimulation, suggesting that the kinase activity of Gsk3β is required for inhibition of SHP2 in TLR2 signaling. Although this finding is interesting, whether and how the serine/threonine kinase Gsk3β controls tyrosine phosphorylation of SHP2 remains to be investigated. One potential hypothesis is that SHP2 might be a substrate of Gsk3β, thereby facilitating inhibition of tyrosine phosphorylation of SHP2.

Our findings suggest that SHP2 is able to regulate IFN-β transcription through inhibition of the DNA binding activity of IRF1 and IRF8. Previous work has revealed that SHP2 is mainly found in the cytoplasm but is also present in the nucleus, where it regulates target gene activation^17, 46–48^. Moreover, Gsk3β shuttles between the cytoplasm and nucleus^49–52^. Indeed, SHP2 associated with Gsk3β together with IRF-1 and IRF-8 in mammalian cells (Supplementary Fig. S6). Moreover, IRF1 and IRF8 mainly localized to the nucleus (Supplementary Fig. S7). We found that SHP2 translocated into the nucleus upon Pam3CSK4 stimulation, suggesting that these interactions may occur in the nucleus. Previously, it was shown that TLR2 activation induces type I IFN-β expression through IRF7-dependent pathway^13^. However, unlike IRF-1 and IRF-8, IRF-7 could not associate with SHP2 or Gsk3β (Supplementary Fig. S8), suggesting that TLR2 may use different IRFs depending on binding partners or cellular contexts.

It has shown previously that TRAM is required for the production of TLR2-dependent type I IFN response^14, 33, 34^. However, TRIF adaptor is not involved in this pathway, and our results agree with those findings because Pam3CSK4-mediated IFN-β induction was impaired by knockdown of TRAM but not TRIF. In contrast, Nilsen *et al*.^33, 35^ reported that TLR2 ligands can induce IFN-β in a TRAM/TRIF-dependent manner. The reason for this discrepancy still remains unclear, but presumably relates to varied experimental settings or conditions.

In conclusion, our results demonstrate that SHP2 and Gsk3β reciprocally regulate type I IFN-β expression in response to TLR2 stimulation. In the context of bacterial infection, it is difficult to provide a simple conclusion regarding the function of type I IFN-β. It appears, instead, that in response to bacteria, type I IFN-β may serve a variety of beneficial and detrimental immune-related functions, many of which remain to be fully understood.

## Methods

### Mice and cell culture

Normal 5- to 8-wk-old C57BL/6 male mice were obtained from the Animal Care Committee of Ewha Laboratory Animal Genomics Center. All animal experiments were approved by the Institutional Animal Care and Use Committee of Ewha Laboratory Animal Genomics Center, and were carried out in accordance with the approved guidelines. Bone marrow-derived macrophages (BMDMs) were obtained from femurs and tibias of C57BL/6 mice. Bone marrow cells were flushed out from the bone marrow cavity and suspended in Dulbecco’s modified Eagle’s medium (DMEM, Hyclone, Logan, UT, USA) supplemented with 20% heat-inactivated fetal bovine serum (FBS, Hyclone), 100 units/ml penicillin (Hyclone) and 100 mg/ml streptomycin (Hyclone). After 1 day in culture, non-adherent cells were cultured in the presence of 10 ng/ml recombinant human M-CSF (R&D Systems, Minneapolis, MN) for 7 days, as described previously^45^. HEK293-Null and TLR2 cells were provided by Dr. S.K. Seo (Inje University, Busan, Korea). Cells were cultured in DMEM supplemented with 10% FBS, 100 units/ml penicillin and 100 mg/ml streptomycin. HEK293-Null and TLR2 cells were transfected with Lipofectamine 2000 (Invitrogen, Paisley, Scotland, U.K.) according to the manufacturer’s instructions.

### Reagents and antibodies

The TLR2 agonist Pam3CSK4 was purchased from Invivogen (San Diego, CA, USA). SB216763, Brefeldin A, and Bafilomycin A1 were purchased from Sigma (St. Louis, MO, USA). NSC87877 was purchased from Santa Cruz Biotechnology (Santa Cruz, CA, USA). Antibodies specific to phosphor-SHP2 (Y542), phosphor-p65 (Y536), phosphor-STAT1 (Y701), Gsk3β, p65, IRF1, IRF8, and HA were purchased from Cell Signaling Technology (Beverly, MA, USA). Antibodies specific to SHP2, IRF8/ICSBP (anti-goat), GAPDH, and β-actin were purchased from Santa Cruz Biotechnology. The antibody against Gsk3α/β was purchased from Invitrogen Life Technologies (Paisley, Scotland, U.K.).

### Plasmids

Wild-type and C463S SHP2 plasmids were obtained from Dr. X. Cao (Chinese Academy of Medical Sciences, Beijing, China). SHP2 E76K was made by performing site-directed mutagenesis. To generate the IRF1 plasmid, an IRF1 DNA fragment was obtained from cDNA isolated from mouse BMDMs by PCR and cloned into the pcDNA3.1-HA expression vector. The IRF8 plasmid was provided by Dr. H. Xiong (Mount Sinai School of Medicine, New York, USA). The IRF8 DNA fragment produced by PCR was cloned into the pEBG expression vector. Plasmids containing various mutants of Gsk3β were described previously^44^. The IFN-β luciferase reporter construct was a gift from Dr. X. Lei (Chinese Academy of Medical Sciences, Beijing, China).

### Quantitative real-time PCR

Total RNA was extracted from cultured cells using TRIzol (Invitrogen). First strand complementary DNA (cDNA) was transcribed using the SuperScript Reverse Transcriptase kit (Invitrogen) from mRNA. Real-time PCR was performed using the KAPA SYBR green FAST qPCR kit (Kapa Biosystems, Boston, MA, USA) on an ABI 7300 real-time PCR machine (Applied Biosystems, Foster City, CA). Data were normalized to β-actin mRNA expression. The gene-specific primers used for real-time PCR were as follows: β-actin, sense; 5′-AGATGTGGATCAAGCAG-3′ and antisense; 5′-GCGCAAGTTAGGTTTTGTCA-3′, IFN-β, sense; 5′-CATCAACTATAAGCAGCTCCA-3′ and antisense; 5′-TTCAAGTGGAGAGCAGTTGAG-3′. Data were normalized to β-actin mRNA expression.

### Retroviral infection

To prepare retroviruses, the Platinum-E (Plat-E) packaging cell line was transfected with various pMX-puro construct DNAs with Lipofectamine 2000 (Invitrogen). The retroviruses were used to infect BMDMs as previously described^53^. The pMX-puro vector and Plat-E cells were provided by T. Kitamura (University of Tokyo). After retroviral infection, the BMDMs were cultured in the presence of M-CSF (10 ng/ml) and puromycine (2 μg/ml) for 2 days. Puromycine-resistant BMDMs were starved with DMEM supplemented with 0.1% FBS (Hyclone) for 3 h, stimulated with Pam3CSK4 for the indicated times, and used for further analysis.

### Immunoprecipitation

Cells were lysed on ice in lysis buffer containing 20 mM HEPES (pH 7.0), 150 mM NaCl, 1% Triton X-100, 10% glycerol, supplemented with protease inhibitors (1 mM PMSF and 1 μg/ml leupeptin and aprotinin) and phosphatase inhibitors (1 mM NaVO4 and 1 mM NaF). For immunoprecipitation, cell lysates were incubated with the indicated primary antibodies at 4 °C for O/N, and were further incubated with protein A- or G-agarose (Millipore, Billerica, MA, USA) at 4 °C for 1 h with rotation. After washing five times with lysis buffer, immunoprecipitated proteins were boiled with 2 × SDS loading buffer, and separated by SDS-polyacrylamide gels.

### Western blot analysis

Cell were lysed in a buffer containing 20 mM HEPES (pH 7.0), 150 mM NaCl, 1% Triton X-100, 10% glycerol, proteinase inhibitors (1 mM PMSF and 1 μg/ml leupeptin and aprotinin) and phosphatase inhibitors (1 mM NaVO4 and 1 mM NaF) after vortexing 5 times for 30 min on ice. After 20 min centrifugation, the supernatants were boiled in 6X SDS sample buffer containing 0.6 M DTT. Cell lysates or immunoprecipitated proteins were separated by 10% SDS-polyacrylamide gels and electrotransferred to a PVDF membrane (Millipore, Billerica, MA, USA). The membranes were blocked with 5% bovine serum albumin (BSA) in Tris-buffered saline containing 0.1% Tween-20 and were immunoblotted with the indicated primary antibodies and secondary antibodies conjugated to HRP. Proteins were detected using an ECL detection kit (Amersham Biosciences, NJ, USA).

### siRNA preparation and transfection

siRNA against mouse negative control, SHP2, IRF1, and IRF8 was synthesized by Genolution (Seoul, Korea). The siRNAs were transfected into BMDMs using the Lipofectamine RNAiMAX reagent (Invitrogen) according to the manufacturer’s instructions. The sequences of the siRNAs were as follows: si-SHP2, CACTGGGGACTACTATGACTT; si-IRF1, GAAGATAGCCGAAGACCTTTT; si-IRF8, ACTCATTCTGGTGCAGGTATT; scrambled nontargeting siRNA, CCTCGTGCCGTTCCATCAGGTAGTT, as a negative control.

### Luciferase assay

HEK293-Null and TLR2 cells were transfected with the IFN-β luciferase reporter constructs and the indicated combinations of gene constructs with Lipofectamine 2000 (Invitrogen) according to the manufacturer’s instructions. After 48 h, the cells were stimulated with Pam3CSK4 (1 μg/ml) and lysed in reporter lysis buffer (Promega, Madison, MI, USA). Luciferase activity was measured with a dual-luciferase reporter assay system (Promega) according to the manufacturer’s instruction. The firefly luciferase activity of samples was normalized to Renilla luciferase activity.

### ChIP assay

This assay was performed with the protocol recommended by Millipore. In brief, BMDMs were cross-linked with 1% formaldehyde for 10 min at 37 °C. Cells were lysed in 200 μl of lysis buffer and sonicated on ice with a Branson Digital Sonifier to shear DNA into pieces with an average size of 500 bp. After centrifugation, supernatants were pre-cleared with a 50% salmon sperm DNA/protein A agarose slurry for 1 h. Chromatin was incubated with 2 μg of normal IgG or anti-IRF1 and IRF8 antibodies O/N with rotation. This was followed by incubation with salmon sperm DNA/protein A agarose slurry for 1 h, then samples were sequentially washed with low salt buffer, high salt buffer, LiCl buffer, and twice with TE buffer. After elution, complexes were reverse cross-linked with NaCl for 4 h at 65 °C and digested with proteinase K, EDTA and Tris-HCl for 1 h at 45 °C. Purified DNA was subjected to quantitative real-time PCR with primers. Input DNA (1%) was used for normalization.

### Statistics

Data are expressed as the mean ± standard deviation (SD) of at least three independent experiments. Statistical analyses were performed using Student’s *t*-test to analyze differences among groups. *P < 0.05 and **P < 0.01 were considered statistically significant.

## Electronic supplementary material

Supplementary Information
