# Supplementary material for: Reciprocal regulation of TLR2-mediated IFN-β production by SHP2 and Gsk3β
Source: Sci Rep. 2017 Jul 28;7:6807. doi: 10.1038/s41598-017-07316-3 (PMC5533723; doi:10.1038/s41598-017-07316-3)
Supplement: Supplementary file 1 — Supplementary Information [file 41598_2017_7316_MOESM1_ESM.pdf]

Supplementary Information

Reciprocal regulation of TLR2-mediated IFN-β production by SHP2 and Gsk3β

Jin Hee Park[1,2], Ryeojin Ko[1,2], and Soo Young Lee[1,2,*]

[1]Department of Life Science, Ewha Womans University, Seoul 120-750, Korea.
[2]The Research Center for Cellular Homeostasis, Ewha Womans University, Seoul 120-750, Korea

[*]Correspondence should be addressed to S.Y.L.

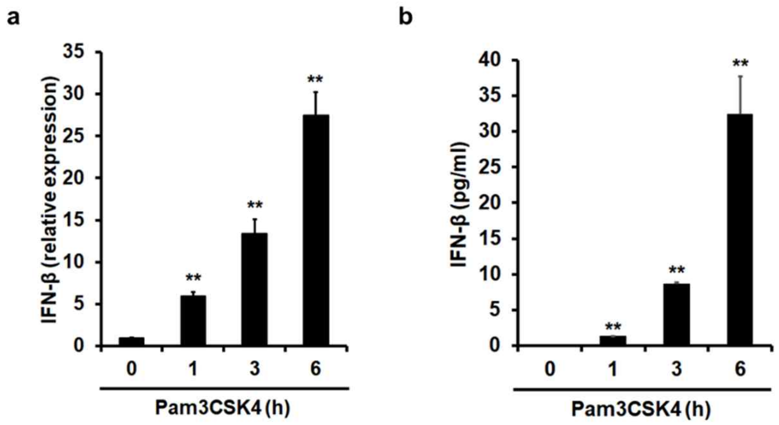

Supplementary Figure S1. IFN-β is produced in BMDMs stimulated with TLR2 ligand. (a, b) BMDMs were stimulated by 1 μg/ml Pam3CSK4 for the indicated times. IFN-β mRNA expression were evaluated by real-time PCR (a). Protein levels of IFN-β were measured by ELISA (b). Data represent the means of triplicate samples ±SD and are representative of at least three experiments. Statistical analyses were calculated using the Student's *t*-test (*P < 0.05, **P < 0.01).

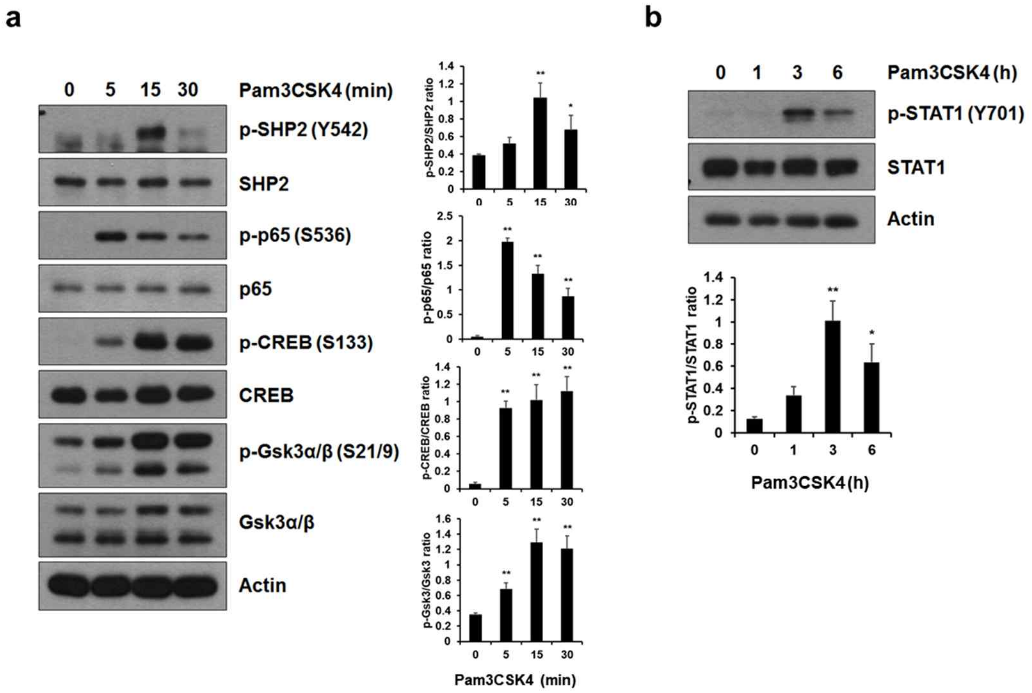

Supplementary Figure S2. TLR2 stimulation activates various signaling pathways. (a) BMDMs were stimulated by 1 μg/ml Pam3CSK4 for the indicated times. Phosphorylation or expression levels of the indicated genes were detected by western blotting. (b) BMDMs were treated with 1 μg/ml Pam3CSK4 for the indicated times. STAT1 phosphorylation and expression levels were determined as described in a. Representative Western blots and quantification (shown in the bar graph) of indicated proteins/control ratio in the lysates of cells are shown in (a, b). Data represent the means of triplicate samples ±SD and are representative of at least three experiments. Statistical analyses were calculated using the Student's *t*-test (*P < 0.05, **P < 0.01). The uncropped images are in Supplementary Fig. S14.

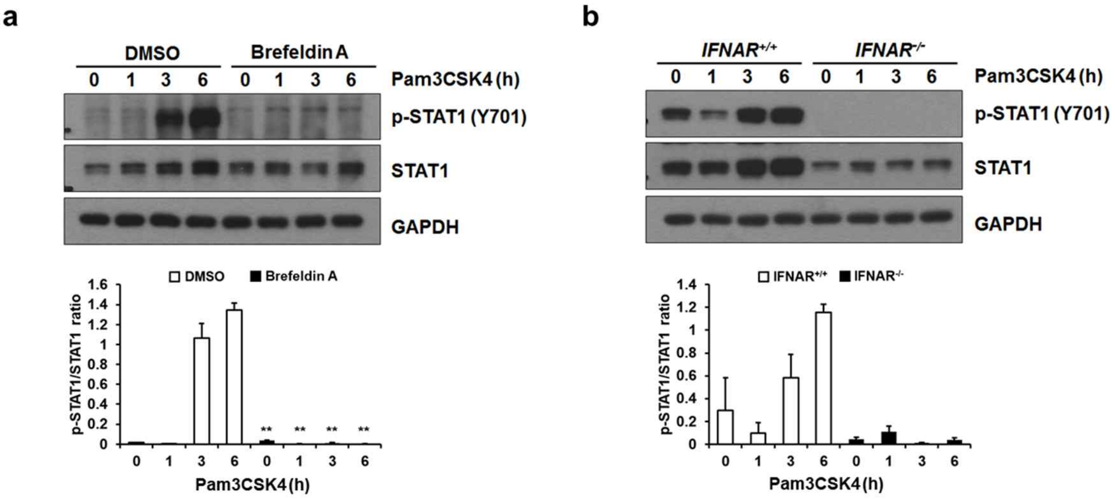

Supplementary Figure S3. Activation of STAT1 in mediated through *INFAR*. (a) BMDMs were pretreated with 1 μg/ml Brefeldin A for 30 min before 1 μg/ml Pam3CSK4 treatment, and STAT1 phosphorylation and expression levels were determined by western blotting. (b) BMDMs from $IFNAR^{+/+}$ and $IFNAR^{-/-}$ mice were treated with 1 μg/ml Pam3CSK4 for the indicated times. Phosphorylation and expression levels of STAT1 were determined as described in a. Representative Western blots and quantification (shown in the bar graph) of indicated proteins/control ratio in the lysates of cells are shown in (a, b). Data represent the means of triplicate samples ±SD and are representative of at least three experiments. Statistical analyses were calculated using the Student's *t*-test (*P < 0.05, **P < 0.01). The uncropped images are in Supplementary Fig. S15.

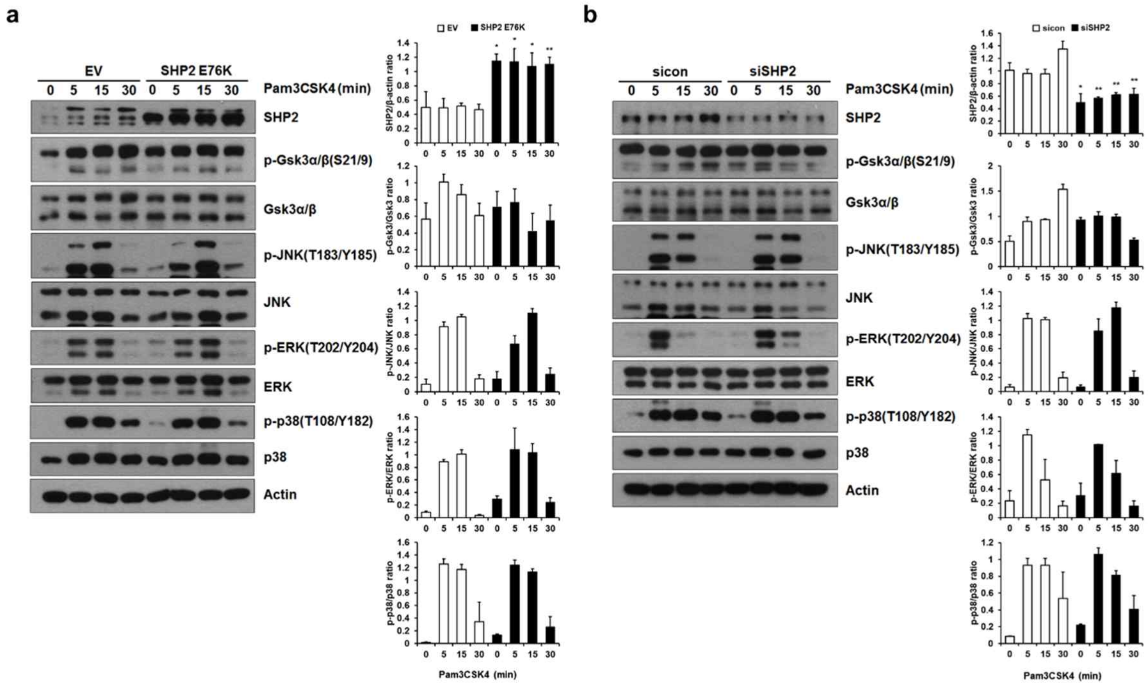

Supplementary Figure S4. SHP2 does not alter Gsk3β activity. (a) BMDMs were infected with retrovirus expressing pMX-Puro (EV) or constitutively active SHP2 (SHP2 E76K). After puromycin selection, 1 μg/ml Pam3CSK4 was treated for the indicated times. Phosphorylation or expression levels of the indicated genes were detected by western blotting. (b) BMDMs were transfected with 50 nM control siRNAs (sicon) or SHP2-specific siRNAs (siSHP2) and stimulated with 1 μg/ml Pam3CSK4 for the indicated times. Phosphorylation or expression levels of the indicated genes were detected as described in a. Representative Western blots and quantification (shown in the bar graph) of indicated proteins/control ratio in the lysates of cells are shown in (a, b). Data represent the means of triplicate samples ±SD and are representative of at least three experiments. Statistical analyses were calculated using the Student's *t*-test (*$P < 0.05$, **$P < 0.01$). The uncropped images are in Supplementary Fig. S16.

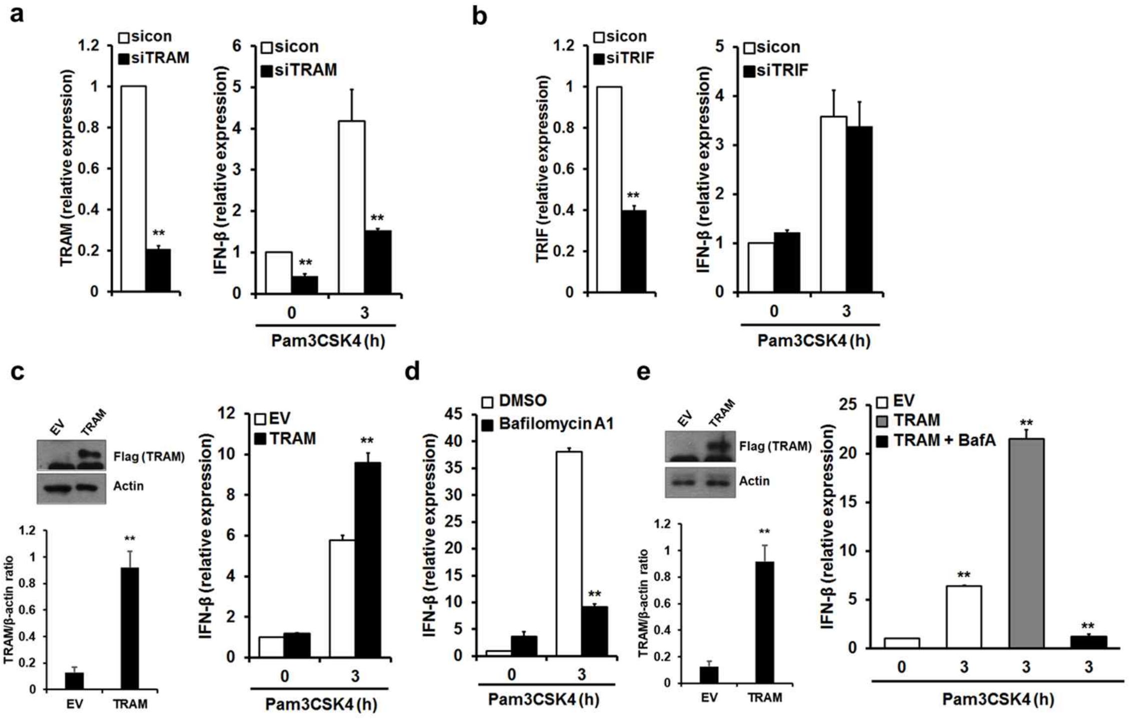

Supplementary Figure S5. TLR2-induced IFN-β production is dependent on TRAM-mediated endocytosis of TLR2. (a, b) BMDMs were transfected with 50 nM control siRNAs (sicon), TRAM- or TRIF-specific siRNAs (siTRAM or siTRIF) and stimulated with 1 μg/ml Pam3CSK4 for 3 h. IFN-β, TRAM and TRIF mRNA expression were evaluated by real-time PCR. (c) BMDMs were infected with retrovirus expressing pMX-Puro (EV) or TRAM. After puromycin selection, cells were treated with 1 μg/ml Pam3CSK4 for 3 h. The mRNA expression of IFN-β was measured as described in a. Expression level of TRAM was detected by western blotting using α-Flag antibody. (d) BMDMs were pretreated with 50 nM Bafilomycin A1 (BafA) for 30 min before 1 μg/ml Pam3CSK4 treatment. mRNA expression of IFN-β in BMDMs was measured as described in a. (e) BMDMs were infected as described in c. After puromycin selection, cells were pre-incubated with 50 nM Bafilomycin A1 (BafA) for 30 min before 1 μg/ml Pam3CSK4 treatment for 3 h. Expression level of TRAM and mRNA expression of IFN-β were measured as described in c. Representative Western blots and quantification (shown in the bar graph) of indicated proteins/control ratio in the lysates of cells are shown in (c, e). Data represent the means of triplicate samples ±SD and are representative of at least three experiments. Statistical analyses were calculated using the Student's *t*-test (*P < 0.05, **P < 0.01). The uncropped images are in Supplementary Fig. S17.

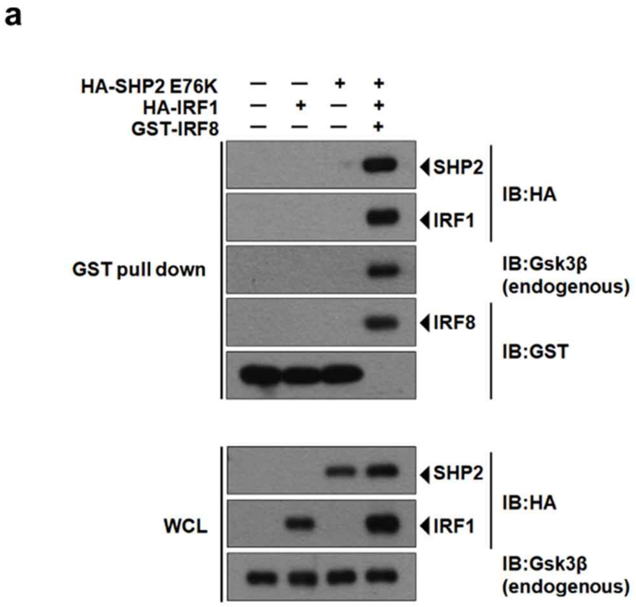

Supplementary Figure S6. IRF8 interacts with IRF1, SHP2 and Gsk3β. (a) HEK293T cells were cotransfected with pEBG-IRF8, constitutively active SHP2 (SHP2 E76K) and HA-IRF1. After 48 h, transfected cells were subjected to GST pull-down assay and analyzed by western blotting using specific antibodies. Data are representative of at least three experiments. The uncropped images are in Supplementary Fig. S18.

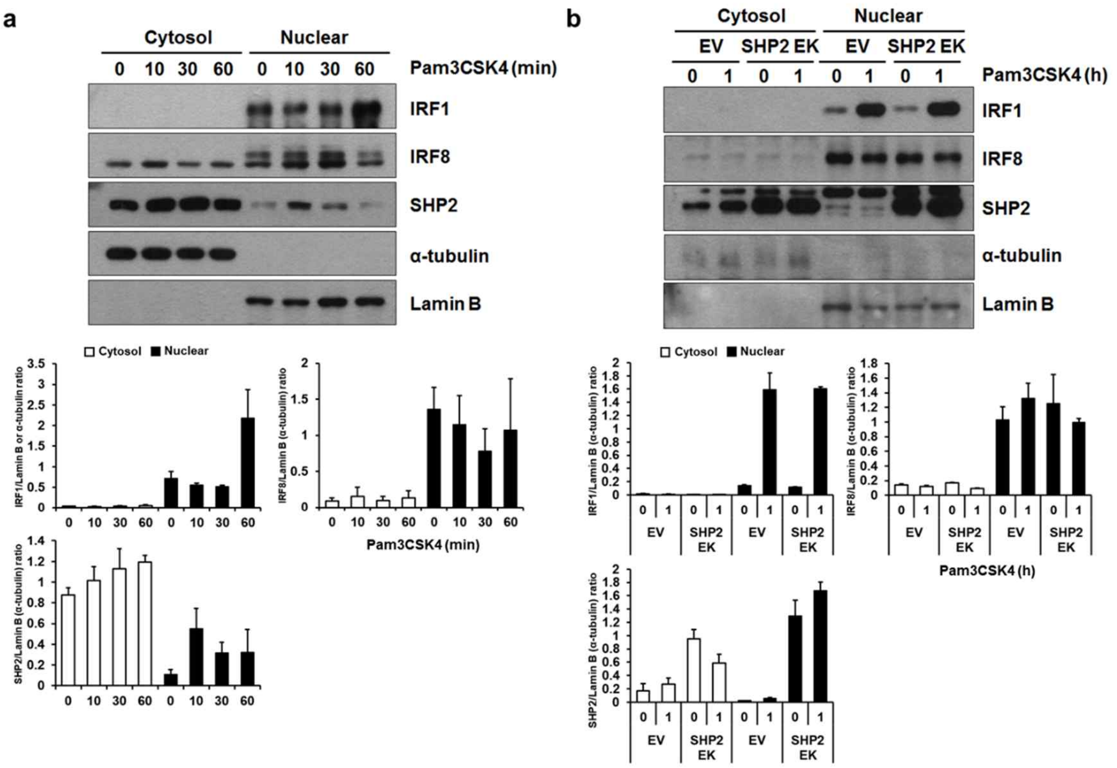

Supplementary Figure S7. SHP2 is not involved in IRF1 or IRF8 nuclear translocation. (a) BMDMs were stimulated with 1 μg/ml Pam3CSK4 for indicated times and cells were separated into cytosolic and nuclear fractions. Indicated gene levels were analyzed by western blotting with specific antibodies. (b) BMDMs were infected with retrovirus expressing pMX-Puro (EV) or constitutively active SHP2 (SHP2 E76K). After puromycin selection, 1 μg/ml Pam3CSK4 was applied for 1 h. Expression levels of cytosolic and nuclear genes were determined as described in a. Representative Western blots and quantification (shown in the bar graph) of indicated proteins/control ratio in the lysates of cells are shown in (a, b). Data represent the means of triplicate samples ±SD and are representative of at least three experiments. Statistical analyses were calculated using the Student's *t*-test (*P < 0.05, **P < 0.01). The uncropped images are in Supplementary Fig. S19.

a

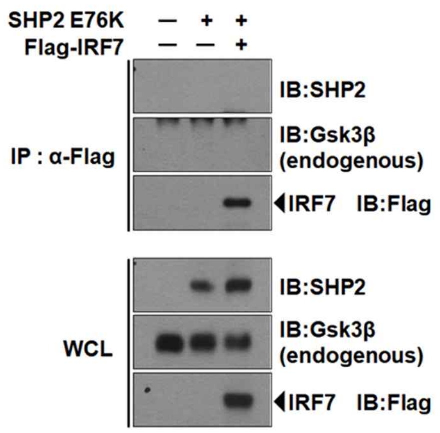

Supplementary Figure S8. IRF7 does not interact with SHP2 and Gsk3β. (a) HEK293T cells were cotransfected with Flag-IRF7 and constitutively active SHP2 (SHP2 E76K). After 48 h, transfected cells were subjected to Co-immunoprecipitation and analyzed by western blotting using specific antibodies. Data are representative of at least three experiments. The uncropped images are in Supplementary Fig. S20.

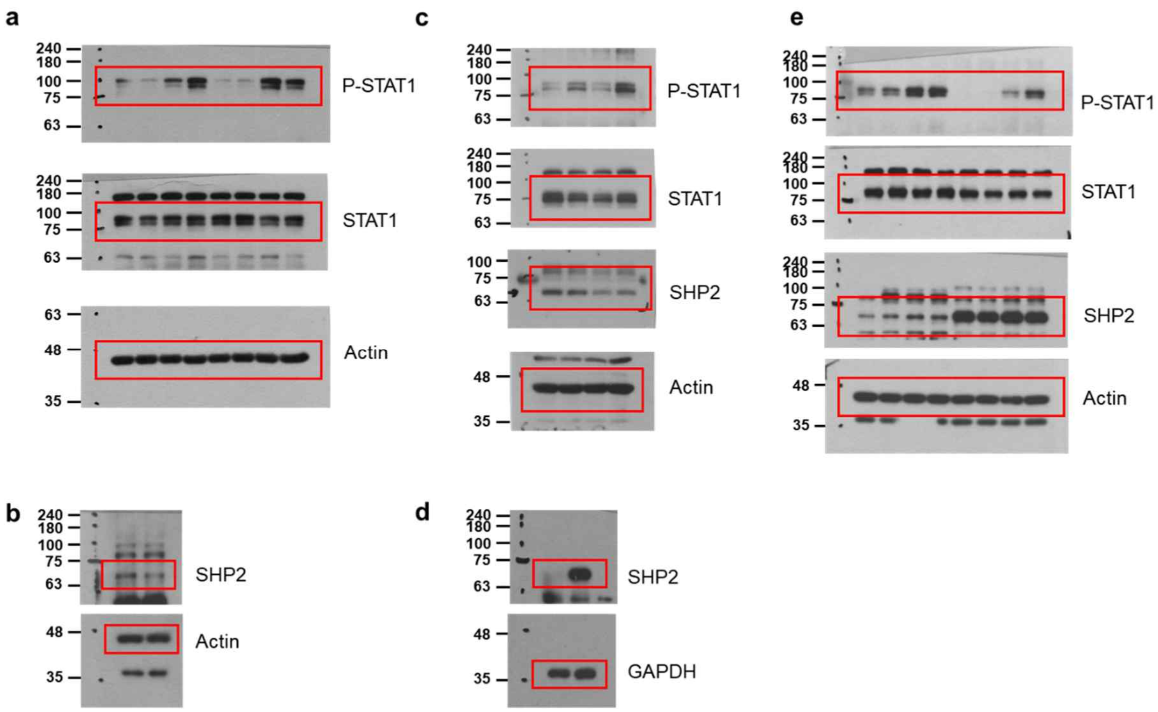

Supplementary Figure S9. Uncropped blots of Figure 1. (a) Figure 1b. (b) Figure 1c. (C) Figure 1d. (d) Figure 1e. (e) Figure 1f.

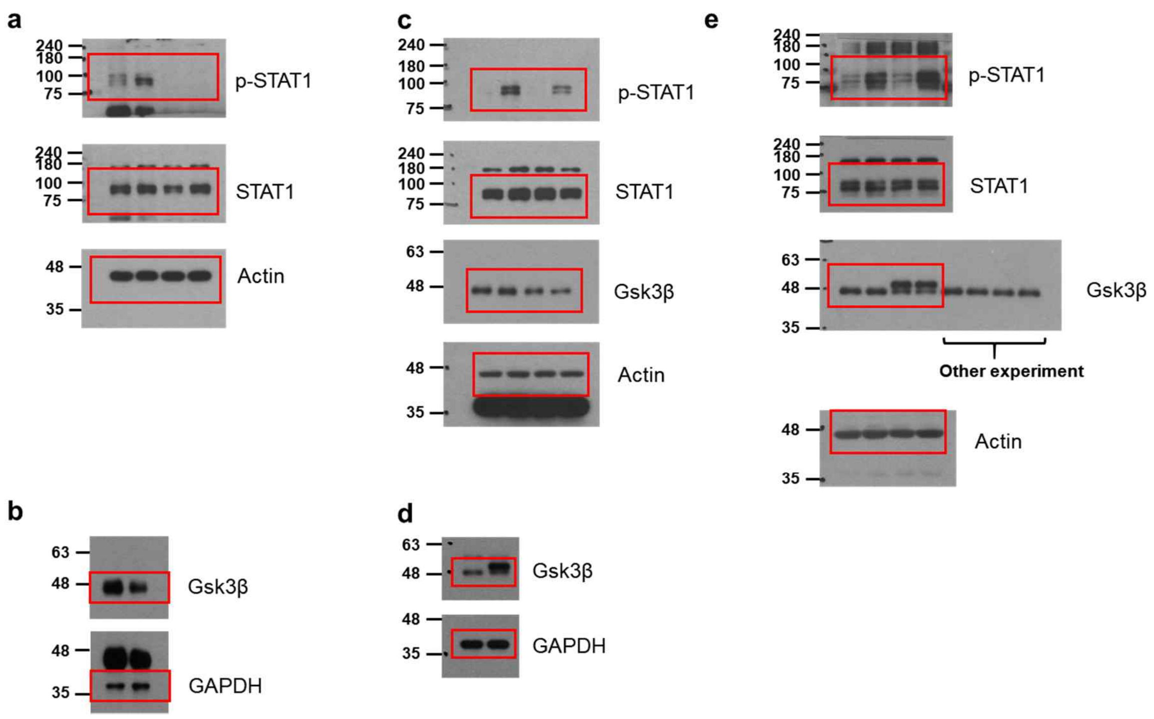

Supplementary Figure S10. Uncropped blots of Figure 2. (a) Figure 2b. (b) Figure 2c. (c) Figure 2d. (d) Figure 2e. (e) Figure 2f.

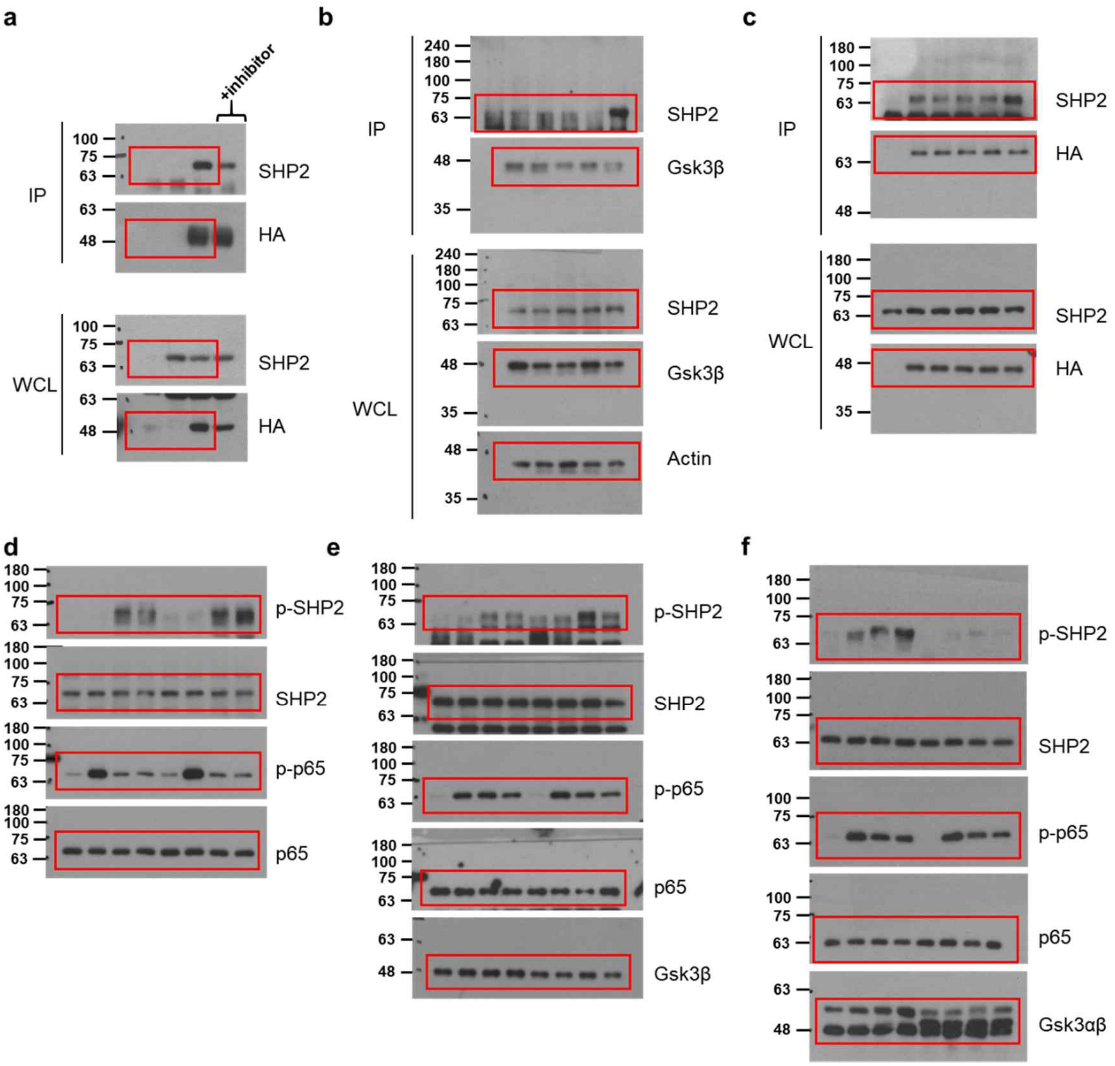

Supplementary Figure S11. Uncropped blots of Figure 3. (a) Figure 3a. (b) Figure 3b. (c) Figure 3c. (d) Figure 3d. (e) Figure 3e. (f) Figure 3f

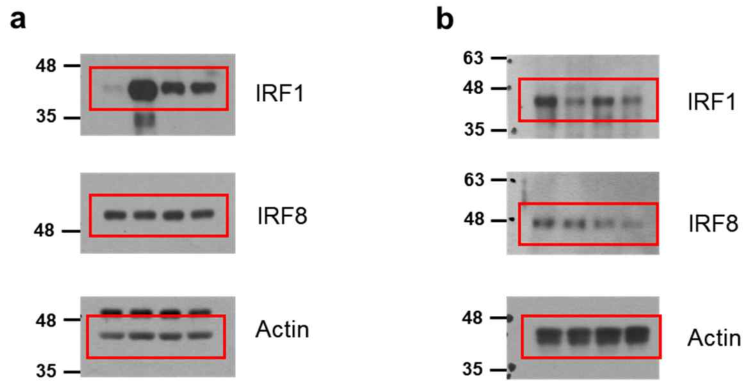

Supplementary Figure S12. Uncropped blots of Figure 4. (a) Figure 4a. (b) Figure 4b.

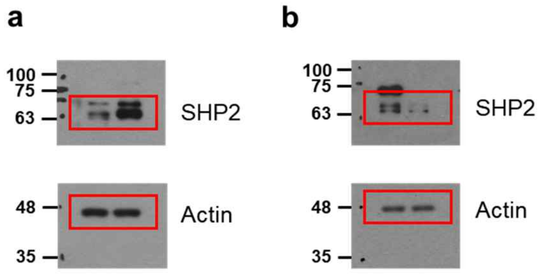

Supplementary Figure S13. Uncropped blots of Figure 5. (a) Figure 5c. (b) Figure 5d.

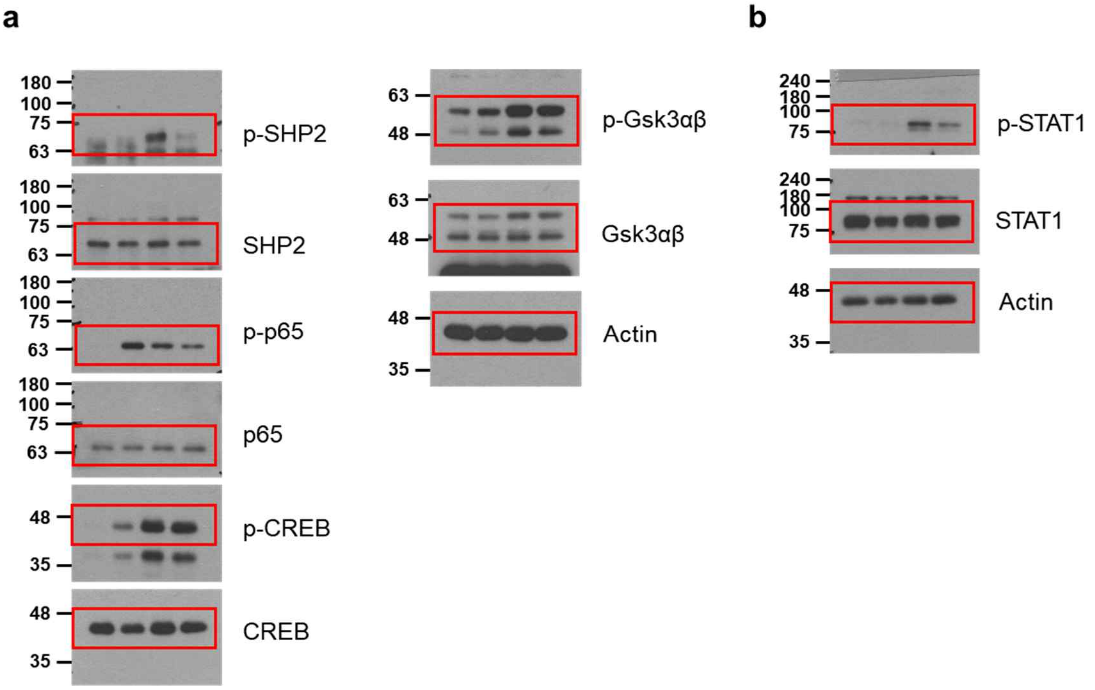

Supplementary Figure S14. Uncropped blots of Supplementary Figure S2. (a) Supplementary Fig. S2a. (b) Supplementary Fig. S2b.

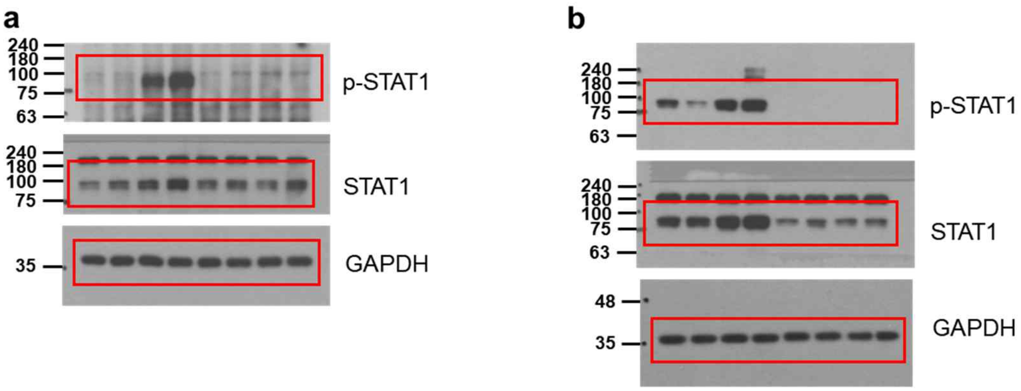

Supplementary Figure S15. Uncropped blots of Supplementary Figure S3. (a) Supplementary Fig. S3a. (b) Supplementary Fig. S3b.

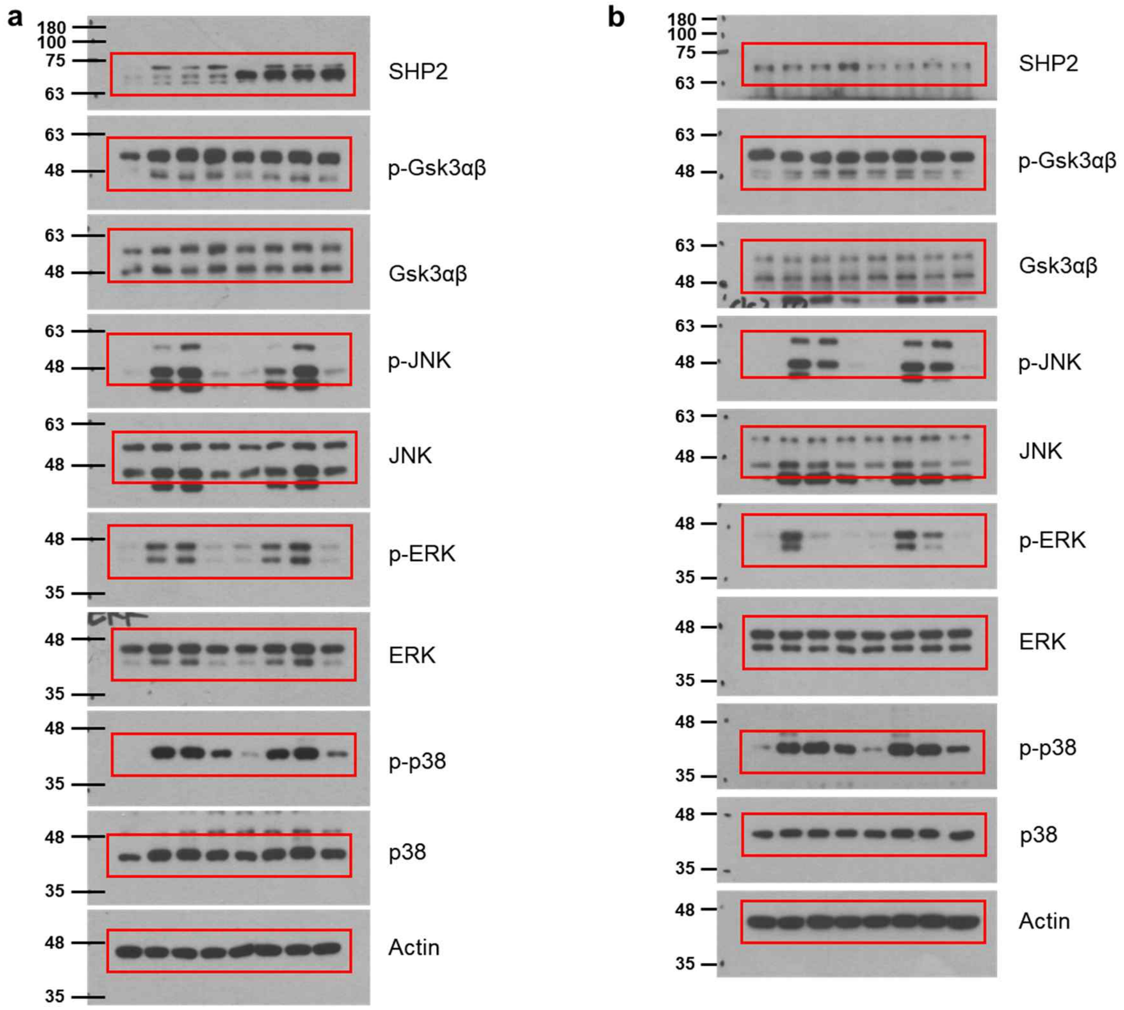

Supplementary Figure S16. Uncropped blots of Supplementary Figure S4. (a) Supplementary Fig. S4a. (b) Supplementary Fig. S4b.

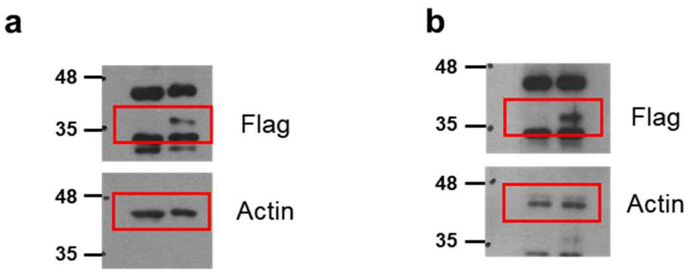

Supplementary Figure S17. Uncropped blots of Supplementary Figure S5. (a) Supplementary Fig. S5c. (b) Supplementary Fig. S5e.

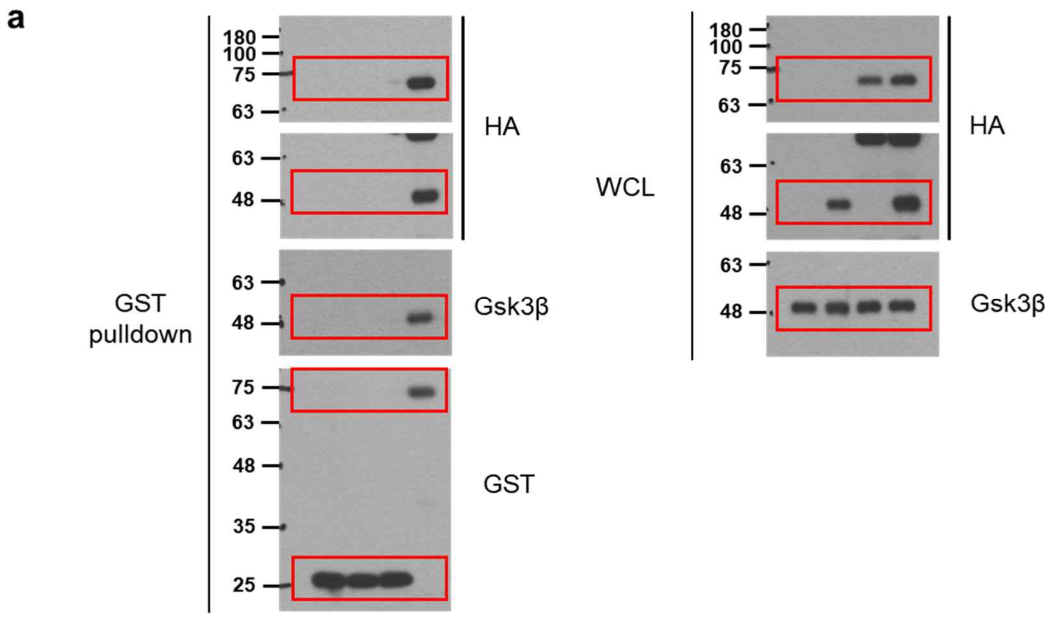

Supplementary Figure S18. Uncropped blots of Supplementary Figure S6. (a) Supplementary Fig. S6a.

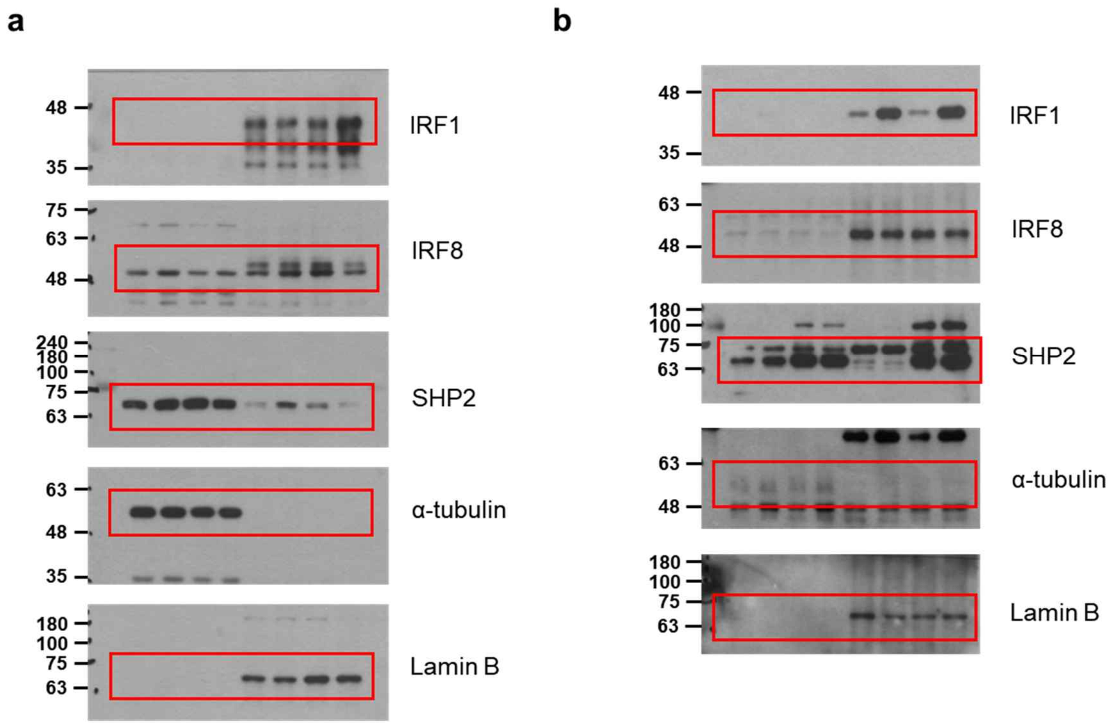

Supplementary Figure S19. Uncropped blots of Supplementary Figure S7. (a) Supplementary Fig. S7a. (b) Supplementary Fig. S7b.

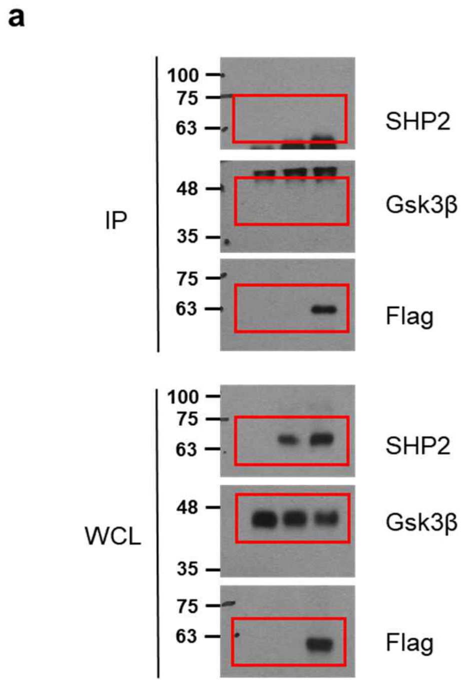

Supplementary Figure S20. Uncropped blots of Supplementary Figure S8. (a) Supplementary Fig. S8a.

Supplementary methods

Enzyme-linked immunosorbent assay. To measure mouse IFN-β levels, BMDMs were starved for 3 h and then stimulated with or without 1 μg/ml Pam3CSK4 for indicated times. Cell culture supernatants were assessed using ELISA kits from PBL assay science (Piscataway, NJ, USA) according to the manufacturer's instructions.

Quantitative real-time PCR. Total RNA was extracted from cultured cells using TRIzol (Invitrogen). First strand complementary DNA (cDNA) was transcribed using SuperScript Reverse Transcriptase kit (Invitrogen) from mRNA. Real-time PCR was performed by KAPA SYBR green FAST qPCR kit (Kapa Biosystems, Boston, MA, USA) on an ABI 7300 real-time PCR machine (Applied Biosystems, Foster City, CA). Data were normalized to β-actin mRNA expression. The gene-specific primers for the real-itme PCR were as follows: mTRAM sense; 5'-CGATCAAGACGGCCATGAGTC-3' and antisense; 5'-CTCGTCGGTGTCATCTTCTGC-3', mTRIF sense; 5'-ATGGGCCCAGCAAGCTATGTAAC-3' and antisense; 5'-AGGGGAGGCTTGGAGGGATGGT-3'. Data were normalized to β-actin mRNA expression.

siRNA preparation and transfection. Small interfering RNA (siRNA) to target mouse negative control, TRAM and TRIF was synthesized from Genolution (Seoul, Republic of Korea). The siRNAs were transfected into BMDMs using the Lipofectamine RNAiMAX reagent (Invitrogen), according to the manufacturer's instructions. Sequences for the siRNAs were as follows: si-mTRAM, CGAGATGCCTGCGGAAGATT; si-mTRIF, GCTATGTAACACACCGCTGTT; scrambled nontargeting siRNA, CCTCGTGCCGTTCCATCAGGTAGTT, as a negative control.

Retroviral infection. To prepare retroviruses, the Platinum-E (Plat-E) packaging cell lines were transfected with pMX-puro-SHP2 E76K and mTRAM DNAs with Lipofectamine 2000 (Invitrogen). The retroviruses were used to infect BMDMs. After retroviral infection, the BMDMs were cultured in the presence of M-CSF (10 ng/ml) and puromycine (2 μg/ml) for 2 days. Puromycine-resistant BMDMs were starved with DMEM supplemented with 0.1% FBS (Hyclone) for 3 h, stimulated Pam3CSK4 for indicated times and experimented by further analysis.

GST pull-down assay and Immunoprecipitation. For GST-pulldown assay, cells were lysed with lysis buffer containing 50 mM Tris-HCl, pH 8.0, 150 mM NaCl, 0.5% deoxycholate acid, 1% NP-40, and protease inhibitors. Cell lysates were incubated with glutathione-sepharose 4B (GE Healthcare, Piscataway, NJ, USA) at 4 °C for 4 hr with rotation. After washing 5 times with lysis buffer, proteins were boiled with 2×SDS loading buffer. For immunoprecipitation, cells were lysed on ice in

lysis buffer containing 20 mM HEPES (pH 7.0), 150 mM NaCl, 1% Triton X-100, 10% glycerol, supplemented with protease inhibitors (1 mM PMSF and 1 μg/ml leupeptin and aprotinin) and phosphatase inhibitors (1 mM NaVO4 and 1 mM NaF). Cell lysates were incubated with the indicated primary antibodies at 4 °C for O/N, and were further incubated with protein A- or G-agarose (Millipore, Billerica, MA, USA) at 4 °C for 1 h with rotation. After washing five times with lysis buffer, immunoprecipitated proteins were boiled with 2×SDS loading buffer, and separated by SDS-polyacrylamide gels

Western blot analysis. BMDM from *IFNAR*$^{+/+}$ and *IFNAR*$^{-/-}$ provided by Dr. M.S. Kweon (University of Ulsan College of Medicine, Seoul, Republic of Korea) or normal mice and HEK293T cells were lysed in a buffer containing 20 mM HEPES (pH 7.0), 150 mM NaCl, 1% Triton X-100, 10% glycerol, and proteinase inhibitors (1 mM PMSF and 1 μg/ml leupeptin and aprotinin） and phosphatase inhibitors (1 mM NaVO4 and 1 mM NaF) by vortexing 5 times for 30 min on ice. After 20 min centrifugation, the supernatants were boiled in 6X SDS sample buffer containing 0.6 M DTT. Cell lysates were separated by 10% SDS-polyacrylamide gels and electrotransferred to a PVDF membrane (Millipore, Billerica, MA, USA). The membranes were blocked with 5% bovine serum albumin (BSA) in Tris-buffered saline containing 0.1% Tween-20 and were immunoblotted with the indicated primary antibodies and secondary antibodies conjugated to HRP. Proteins were detected using an ECL detection kit (Amersham Biosciences, NJ, USA). The following primary antibodies were used: phosphor-Gsk3αβ (Ser21/S9), phosphor-SHP2 (Y542), phosphor-CREB (Y133), phosphor-p65 (Y536), phosphor-STAT1 (Y701), phospho-p38(T108/Y182), phospho-JNK(T183/Y185), phospho-ERK(T202/Y204), p38, JNK, ERK, Gsk3β, p65, CREB, IRF1, IRF8, HA and SHP2 from Cell signaling, Lamin B, TBP, α-tubulin, GAPDH and β-actin from Santa Cruz Biotechnology, α-Flag from Sigma and Gsk3αβ from Invitrogen.

Cytoplasmic and nuclear fractionation.
BMDMs were lysed with cytosolic fractionation buffer (10 mM KCl, 1.5 mM MgCl2, 10 mM HEPEs, pH 7.4, 0.5 M dithiothreitol, 0.04% NP-40) containing protease inhibitors (1 mM PMSF and 1 μg/ml leupeptin and aprotinin). Lysates were centrifuged at 800g for 10 min at 4 °C and supernatants were used for the cytosolic fraction. Pellets were washed with cytosolic fractionation buffer and lysed with nuclear fractionation buffer (300 mM NaCl, 1.5 mM MgCl2, 5 mM HEPES, pH 7.4, 0.2 mM EDTA, 25% glycerol) containing protease inhibitors and then incubated for 30 min on ice with vortexing three times. After centrifugation at 14,000 r.p.m for 30 min at 4 °C, the supernatants were used for the nuclear fraction.
